# Supplementary material for: Splice-Junction-Based Mapping of Alternative Isoforms in the Human Proteome
Source: Cell Rep. Author manuscript; Available in PMC 2020 Jan 15. (PMC6961840; doi:10.1016/j.celrep.2019.11.026)
Supplement: 2 [file NIHMS1546469-supplement-2.zip › DF1/PXD006675/Aorta/Aorta_8_HMGB1_ENILACPLVMLR.pdf]

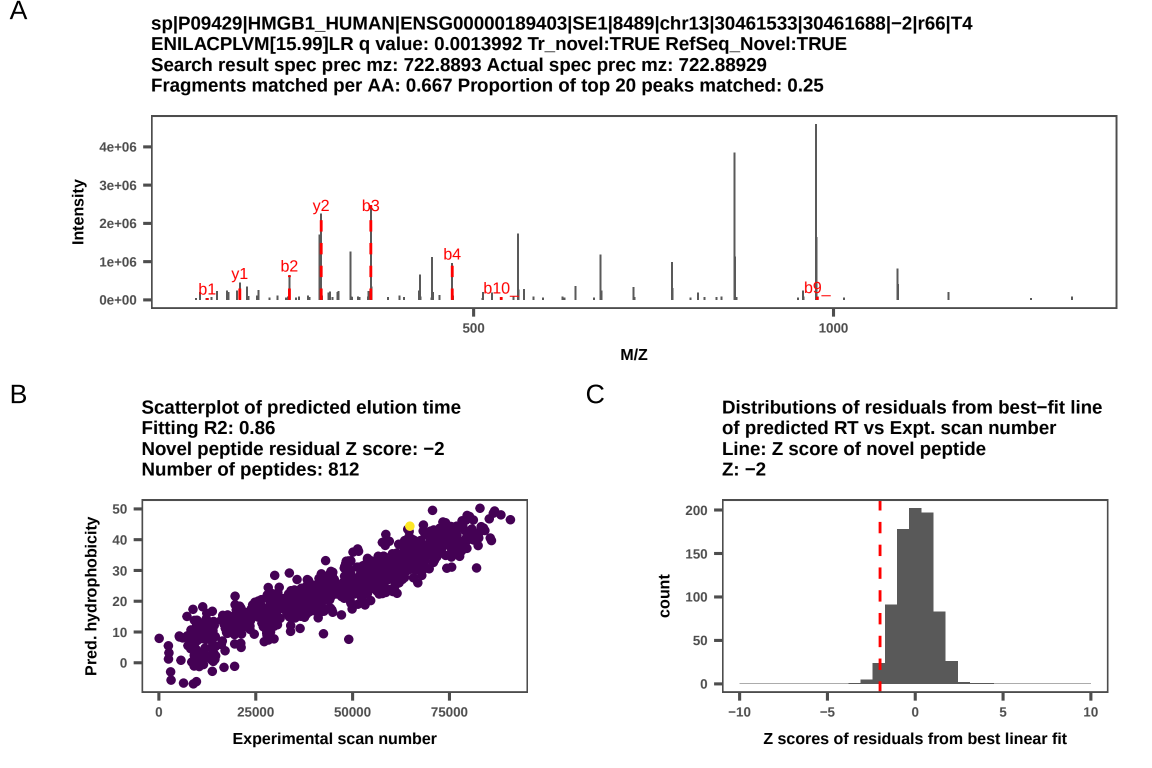
A
sp|P09429|HMGB1_HUMAN|ENSG00000189403|SE1|8489|chr13|30461533|30461688|−2|r66|T4
ENILACPLVM[15.99]LR q value: 0.0013992 Tr_novel:TRUE RefSeq_Novel:TRUE
Search result spec prec mz: 722.8893 Actual spec prec mz: 722.88929
Fragments matched per AA: 0.667 Proportion of top 20 peaks matched: 0.25
Intensity
4e+06
3e+06
2e+06
1e+06
0e+00
b1
y1
b2
y2
b3
b4
b10_
b9_
500
1000
M/Z
B
Scatterplot of predicted elution time
Fitting R2: 0.86
Novel peptide residual Z score: −2
Number of peptides: 812
Pred. hydrophobicity
50
40
30
20
10
0
0
25000
50000
75000
Experimental scan number
C
Distributions of residuals from best−fit line
of predicted RT vs Expt. scan number
Line: Z score of novel peptide
Z: −2
count
200
150
100
50
0
−10
−5
0
5
10
Z scores of residuals from best linear fit
